# Hemodiafiltration with ultrafiltrate regeneration reduces free light chains without albumin loss in multiple myeloma patients

**DOI:** 10.1186/s12882-020-01885-8

**Published:** 2020-06-15

**Authors:** M. Victoria Pendón-Ruiz de Mier, Raquel Ojeda, M. Antonia Álvarez-Lara, Ana Navas, Corona Alonso, Javier Caballero-Villarraso, Pedro Aljama, Miguel A. Álvarez, Sagrario Soriano, Mariano Rodríguez, Alejandro Martín-Malo

**Affiliations:** 1 Nephrology Service, Reina Sofia University Hospital, Avenida Menéndez Pidal S/N, 14004 Córdoba, Spain; 2 Research Unit, Maimonides Institute for Biomedical Research (IMIBIC), Cordoba, Spain; 3 University of Cordoba, Cordoba, Spain; 4 Spanish Renal Research Network (REDinREN), Institute of Health Carlos III, Madrid, Spain; 5 Quality Service, Reina Sofia University Hospital, Cordoba, Spain; 6 Immunology Service, Reina Sofia University Hospital, Cordoba, Spain; 7 Clinical Analysis Service, Reina Sofia University Hospital, Cordoba, Spain; 8 Hematology Service, Reina Sofia University Hospital, Cordoba, Spain

**Keywords:** Acute kidney failure, Adsorption, Albumin loss, Dialysis, Free light chains, Myeloma

## Abstract

**Background:**

Acute kidney injury (AKI) occurs in 12–20% of multiple myeloma (MM) patients. Several studies have shown a reduction of free light chains (FLC) using hemodialysis with High-Cut-Off membranes. However, this technique entails albumin loss. Hemodiafiltration with ultrafiltrate regeneration is a technique that includes a process of adsorption. The aim of this study was to evaluate the effectiveness of hemodiafiltration with ultrafiltrate regeneration in reducing FLC levels without causing albumin loss.

**Methods:**

This is an observational study (2012 to 2018) including nine patients with MM (5 kappa, 4 lambda) and AKI. All patients were treated with chemotherapy and hemodiafiltration with ultrafiltrate regeneration. Blood Samples (pre and post-dialysis) and ultrafiltrate were collected pre and post-resin at 5 min after initiation of the session and 5 min before the end of the procedure.

**Results:**

The serum levels of kappa and lambda were reduced by a 57.6 ± 10% and 33.5 ± 25% respectively. Serum albumin concentration remained unchanged after the procedure. In the ultrafiltrate, the mean FLC reduction ratio shortly after initiation of the dialysis procedure was: 99.2 and 97.06% for kappa and lambda respectively, and only 0.7% for albumin; and at the end of the session the percent reduction was: 63.7 and 33.62% for kappa and lambda respectively, and 0.015% for albumin. Patients clinical outcome was: 33.3% recovered renal function, 22.2% died during the first year and 44.4% required maintenance dialysis.

**Conclusions:**

Hemodiafiltration with ultrafiltrate regeneration reduces FLC levels without producing a significant loss of albumin; and, FLC removal is maintained throughout the session. Therefore, hemodiafiltration with ultrafiltrate regeneration may be considered an effective adjunctive therapy in patients with MM.

## Background

Multiple myeloma (MM) is a malignant plasma cell proliferation characterized by overproduction of monoclonal immunoglobulins. Severe acute kidney failure (AKI) is observed in 12–20% of MM patients and it is produced mainly by deposition of light chains, cast formation and tubular obstruction (myeloma cast nephropathy) [1, 2]. There have been improvements in the management of this disease, however severe renal failure continues to be an important burden that worsens the prognosis [3]; in fact dialysis is required in a 10% of cases [1, 4, 5].

The production of free light chains (FLC) is reduced with the use of chemotherapy (dexamethasone, bortezomib, melphalan, thalidomide, lenalidomide, cyclophosphamide) associated or not to autotransplantation of hematopoietic cells [6]. An adjuvant therapy is the use of extrarenal depuration techniques to reduce the levels of FLC so the kidney damage is minimized. In recent years, several studies have been published on the effectiveness of very high permeability, “High-Cut-Off” (HCO) membranes for the removal of FLC and protein bound uremic toxins [7]. With a sustained reduction of FLC the recovery of kidney function has been reported to be as high as 64% which is associated to better survival [8–11]. However other authors have not reported a significant difference in renal function recovery between HCO and high flux hemodialysis [12, 13]. An important drawback of procedures using HCO is the abundant loss of albumin requiring continuous replacement [6, 8].

Hemodiafiltration with regeneration of the ultrafiltrate by adsorption using a resin (Supra HFR) has been introduced as an extrarenal clearance technique that combines convection, adsorption and diffusion; it uses a high cut-off “Super High Flux” polyphenylene membrane. We have shown that hemodiafiltration with ultrafiltrate regeneration, may improve uremic protein-bound toxin removal, inflammatory state, endothelial damage, and oxidative stress as compared with on-line hemodiafiltration and high-flux hemodialysis [14]. Since a high cut-off membrane should allow the passage of FLC (especially kappa), without the loss of albumin (molecular weight 60 kD), hemodiafiltration with ultrafiltrate regeneration might be a reasonable strategy for FLC removal [15]. We had explored the feasibility of using hemodiafiltration with ultrafiltrate regeneration in 3 patients with MM and AKI, the results were encouraging and there was no need for albumin replacement [16]. The aim of the present study is to evaluate the effectiveness of hemodiafiltration with ultrafiltrate regeneration in reducing FLC and its effect on albumin in patients with AKI secondary to MM.

## Methods

### Patients

This is an observational study that includes 9 patients with AKI due to MM diagnosed in our centre between July 2012 and December 2018. All patients were immediately treated with chemotherapy according to Hematology protocol (based on bortezomib and corticosteroids) and hemodiafiltration with ultrafiltrate regeneration was used as renal replacement therapy. All patients with AKI and MM requiring renal replacement treatment were dialyzed with Supra HFR, even if serum FLC were < 500 mg/L, the intention was to maintain FLC levels below this threshold. The main criteria for renal replacement therapy was a decreased glomerular filtration rate (< 7–10 ml/min/1.73m^2^). The baseline creatinine before diagnosis of MM was normal. The exclusion criteria from the study were cases of relapse MM and those with AKI on a background of chronic kidney disease. All patients were followed until death or censored at 2019 January 31st if remained alive.

The diagnosis of MM was made based on the presence of clonal bone marrow plasma cells (> 10%), serum and/or urinary monoclonal protein and evidence of end organ damage that can be attributed to a plasma cell proliferative disorder [17]. Free light chains removal is useful in myeloma cast nephropathy as reported by several authors [8, 18]. A kidney biopsy was performed in 3 cases that showed myeloma cast nephropathy. In the other 8 patients, a kidney biopsy could not be performed due to clinical conditions that contraindicated the biopsy procedure. To assess the diagnosis of myeloma cast nephropathy in those patients without kidney biopsy the following criteria have to be fulfilled, according to the International Myeloma Working Group Recommendations: (a) past or new diagnosis of MM, (b) serum FLC measurement > 500 mg/L, (c) albuminuria to proteinuria ratio < 30%, and (d) persistence of AKI after the correction of precipitating factors (hypercalcemia, nephrotoxicity, dehydration) [19]. Two additional patients had the diagnosis of MM and AKI but the FLC levels were below the threshold of 500 mg/L. These two patients required renal replacement treatment so it was elected to initiate hemodiafiltration with ultrafiltrate regeneration assuming that this procedure offered more benefit to the patient that a simple hemodialysis procedure. Nevertheless these two patients were not analyzed as the rest of patients.

**Fig. 1:**
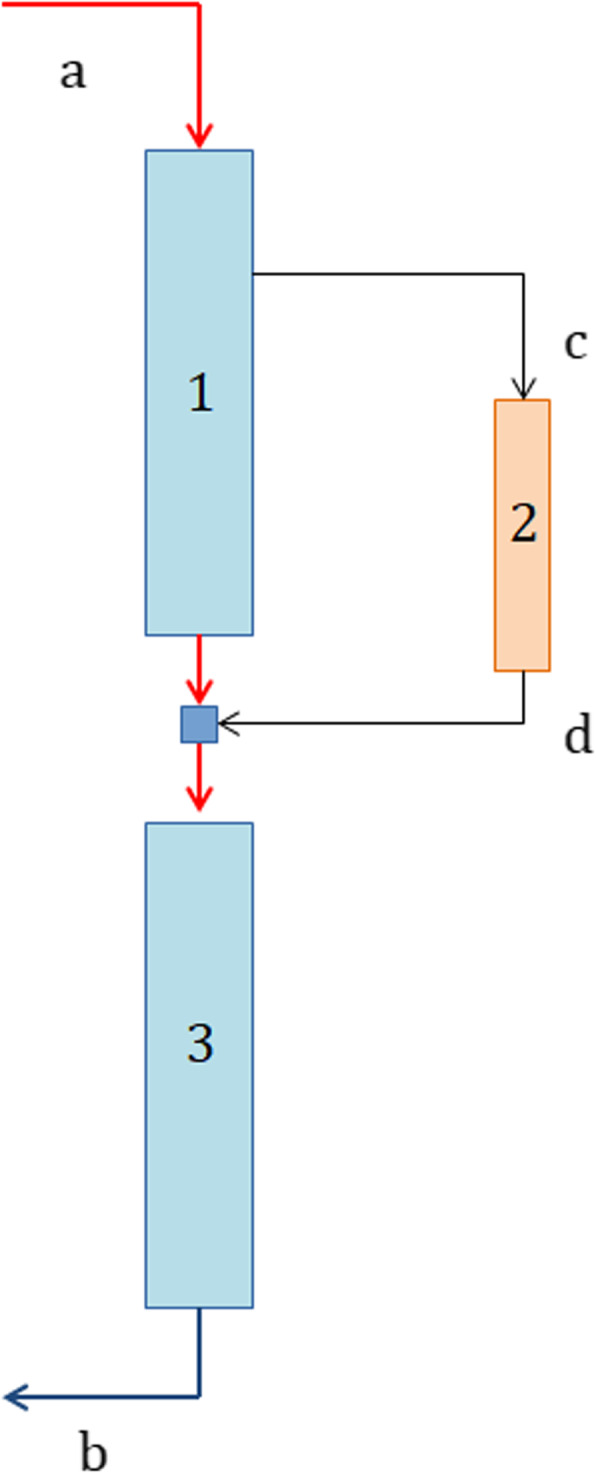
Diagram of hemodiafiltration with regeneration of the ultrafiltrate by adsorption in resin. 1: High permeability filter (Convection). 2: Resin cartridge (Adsorption). 3: Low permeability filter (Diffusion). Protocol of samples extraction: The blood samples were obtained from the arterial port of the central venous catheter before (pre) (**a**) and immediately after (post) (**b**) the completion of the dialysis procedure (a total of 2 samples). Ultrafiltrate, pre (**c**) and post (**d**) resin, samples were taken at 5 min after starting the session (2 samples) and 5 min before the end of the session (2 samples). A total of six samples were collected during the procedure: 2 arterial blood samples and 4 samples of ultrafiltrate.

The procedure used to reduce the circulating levels of FLC was the hemodiafiltration with ultrafiltrate regeneration (Supra HFR) (Bellco©/Medtronic©). This technique combines convection, adsorption and diffusion; this is accomplished by two filters and a cartridge. The convection process takes place in the first filter, a Super High Flux polyphenylene membrane with a high cut-off and a 0.7 m^2^ surface. The generated ultrafiltrate (UF) circulates throughout a cartridge of adsorbent resin (Suprasorb, 80 ml) at a maximum flow rate of 70 ml/min, to remove the uremic toxins from the UF. The UF is subsequently reinfused before it reaches a second chamber containing a low permeability polyphenylene filter of 1.7 m^2^ surface, where the diffusion process takes place. A scheme of the procedure is shown in the Fig. 1.

Blood and dialysate flow were set at 300–350 and 500 ml/min respectively. The net ultrafiltration rate was programmed according to interdialysis weight gain. The dialysate calcium and potassium concentration were 3 and 2 mEq/L respectively. The volume of endogenous UF for each 4-h session was between 13.2–14.4 l. The number of hemodiafiltration with ultrafiltrate regeneration sessions was individually adjusted in each case. The duration of the 2 initial dialysis sessions was 150 and 180 min respectively, the remaining sessions 240 min three times a week. The first 2 dialysis sessions lasted only 150 and 180 min respectively because in the design of this observational study, a daily incremental dialysis dose (150, 180 and 240 min) was prescribed. Anticoagulation was achieved by initial bolus of heparin sodium and an hourly dose. A temporary catheter was placed as an initial vascular access and, after 2 weeks of treatment, it was replaced by a tunneled catheter. Treatment with hemodiafiltration with ultrafiltrate regeneration was extended while the patient required dialysis, even if the serum light chains levels were less than 500 mg/L.

### Laboratory tests

Blood samples were obtained from the blood lines pre- and post- filters at the beginning and at the end of the procedure. Samples of UF were drawn before and after the resin cartridge, 5 min after the initiation and 5 min before the completion of the procedure (Fig. 1). The concentration of FLC and albumin were measured in each sample. The serum levels post-dialysis were corrected by the ultrafiltration rate in each case. The extractions were always obtained in the first weekly dialysis. The samples were stored until measurements of FLC below 500 mg/L were obtained.

Albumin was measured by the bromocresol purple colorimetric enzymatic method. The quantification of kappa and lambda FLC in blood and UF samples was performed with the Freelite kit (The Binding Site Group Ltd.) for use in the Siemens BN® II nephelometer; this is a test validated for renal failure patients [20]. The normal range is 3.3–19.4 mg/L for kappa and 5.71–26.3 mg/L for lambda.

The reduction ratio per session (RRs) was calculated as follows:

$$ \mathrm{RRs}=\left({\mathrm{C}}_{\mathrm{pre}}-{\mathrm{C}}_{\mathrm{post}-\mathrm{corr}}\right)/{\mathrm{C}}_{\mathrm{pre}}\times 100 $$

where C_pre_ is the pre-dialysis concentration and C_post-corr_ is the post-dialysis concentration corrected for hemoconcentration according to the following formula:

$$ {\mathrm{C}}_{\mathrm{post}-\mathrm{corr}}={\mathrm{C}}_{\mathrm{post}}/\left(1+\left(\Delta \mathrm{BW}/\left(0.2\ \mathrm{x}\ {\mathrm{BW}}_{\mathrm{post}}\right)\right)\right) $$

where C_post_ is the post-dialysis concentration without correction, ΔBW is the weight reduction during dialysis (ultrafiltration) and BW_post_ is the body weight post dialysis [21].

### Statistical analysis

Continuous variables are shown as mean (± standard deviation, SD). Categorical variables are presented as percent (%). A *p*-value < 0.05 was considered statistically significant. Statistical analyses were performed using SPSS statistical program (SPSS Inc., Chicago, IL, USA).

## Results

There were nine patients with AKI secondary to MM included in the study. All patients were treated early after diagnosis, with hemodiafiltration and ultrafiltrate regeneration. Clinical and demographic characteristics are shown in Table 1. The AKI class for each patient according with AKIN classification is also shown in Table 1.

**Table 1:** Baseline demographics characteristics at the diagnosis of MM

Patient	Age (years)	Gender	FLC type	FLC levels at the diagnosis of MM (mg/L)	Serum Creatinine concentration at the diagnosis of MM (mmol/L)	AKIN Clasification (stage)
**1**	64	F	κ	18,806	0.62	III
**2**	52	F	κ	6178	1.17	III
**3**	75	F	λ	826	0.81	III
**4**	72	M	κ	11,200	0.97	III
**5**	74	M	λ	1800	0.75	III
**6**	64	M	λ	569	0.99	III
**7**	85	F	κ	28,023	0.56	III
**8**	76	M	κ	5243	0.61	III
**9**	65	F	λ	5852	0.28	III
**MEAN:**	69.6 ± 9.5	5F / 4 M	5κ / 4λ	13,890 ± 9558 (κ) 2262 ± 2452 (λ)	0.75 ± 0.26	III
**10**	78	M	λ	*379*	0.23	III
**11**	75	M	κ	*466*	0.23	III

Legend: *FLC* free light chain, *MM* multiple myeloma, *κ* Kappa, *λ* Lambda, *F* female, *M* male

All patients were Caucasian (4 males and 5 females), mean age: 69.6 ± 9.5 years with a mean serum creatinine of 0.75 ± 0.26 mmol/L. The serum FLC levels were quantified at diagnosis: kappa FLC isotype was present in 5 patients (55.6%) and lambda FLC isotype in 4 patients (44.4%). The mean concentration of light chain kappa was 13,890 ± 9558 mg/L and the mean concentration of light chain lambda was 2262 ± 2452 mg/L. The initial levels of FLC and the mean percent reduction of FLC in each patient are listed in Tables 1 and 2. After 12 and 21 days from the beginning of chemotherapy and extracorporeal removal of FLC, the RR of FLC was 44.2–45.6% for kappa and 40.7–41.5% for lambda. The number of Supra HFR sessions performed for each patient during the first 3 months is shown in Table 2.

**Table 2:** Data on treatment and clinical evolution at first year

Patient	Onset of HFR since diagnosis (days)	Onset of QT since diagnosis (days)	Mean serum FLC reduction (%)	Mean serum Albumin levels pre dialysis (mmol/L)	Mean serum Albumin levels post dialysis (mmol/L)	Number of Supra HFR sessions	Renal recovery	State
**1**	0	7	63	0.52	0.53	40	No	D
**2**	0	0	72	0.37	0.35	36	Yes	A
**3**	3	7	24	–	–	24	Yes	A
**4**	0	4	54	0.43	0.40	36	Yes	A
**5**	0	0	26	0.42	0.41	40	No	D
**6**	0	3	70	–	–	38	No	A
**7**	0	11	47	–	–	38	No	A
**8**	0	0	52	–	–	38	No	A
**9**	3	1	14	–	–	38	No	A
**MEAN:**	0.67 ± 1.32	3.67 ± 3.93	57.6 ± 10 (κ) 33.5 ± 25 (λ)	0.43 ± 0.06	0.42 ± 0.07	36 ± 4		
**10**	0	0	30	0.44	0.39	38	No	A
**11**	0	3	32	0.50	0.49	12	Yes	A

Legend: *HFR* hemodiafiltration with ultrafiltrate regeneration, *QT* chemotherapy, *FLC* free light chain, *A* alive, *D* deceased

Serum albumin concentration pre and post session were similar (0.43 ± 0.06 mmol/L pre vs. 0.42 ± 0.07 mmol/L post). Serum albumin levels were measured in 4 patients and in the two other patients. In the other 5 patients, this measurement was not performed due to practical problems. The FLC removal from the UF by the cartridge at the beginning of the procedure was 99.2% for kappa and 97.06% for lambda light chains; the uptake of albumin was trivial (0.7%). At the end the session the capacity of the cartridge to adsorb kappa light chain was still very significant (63.7%); and the ability to remove lambda light was still sizeable (33.6%). The loss of albumin remained very low at the end of the procedure (0.015%). Moreover, no complications associated with the technique were observed.

In 3 patients (33.3%) (patients 2, 3 and 4) the renal function improved after 2.75 ± 0.43 months of treatment and the mean creatinine decreased from 0.99 ± 0.18 mmol/L to 0.29 ± 0.14 mmol/L; then, it continued to decrease until the end of the follow up (Creatinine: 0.18 ± 0.04 mmol/L). Two patients (22.2%) died during the first year (patients 1 and 5) and 4 patients (44.4%) required maintenance dialysis (patients 6, 7, 8 and 9). Patients 9 and 11 did not complete 1 year of follow-up; patient 11 recovered renal function after 17 days of treatment. With respect to causes of deaths, patient 1, dependent on dialysis, died of septic shock 10 days after an autotransplant of hematopoietic cells (7.5 months after diagnosis of MM). Patient 5, who was also dependent on dialysis, died of septic shock at month 11. Patient 4 recovered renal function but died 7 months later due to a septicemia (his creatinine was 0.22 mmol/L); and patient 10, who was dependent on dialysis, committed suicide at month 16. Two patients died from septic shock due to respiratory infection. The tunneled catheter was not the source of the infection. Moreover, an arteriovenous fistula was created in patients who did not recover renal function after 3 months of treatment.

## Discussion

The present study was performed in patients with AKI secondary to MM to evaluate the effectiveness of hemodiafiltration with ultrafiltrate regeneration in reducing FLC and to determine whether there is albumin loss with this procedure. Our results show that the hemodiafiltration with ultrafiltrate regeneration technique produces an effective and sustained removal of FLC, without a significant loss of albumin. Therefore, this technique is effective as an adjunctive treatment for MM in combination with chemotherapy allowing the renal recovery in 33.3% of patients.

To our knowledge, the present report is the largest series of multiple myeloma patients treated with hemodiafiltration with ultrafiltrate regeneration. There were 11 patients analyzed, which may be sufficient to estimate the percentage reduction of FLC. Kappa and lambda FLC have a molecular weight of 22.5 kD and 45 kD, respectively. Testa et al. showed that this technique removes FLC, particularly kappa, in patients with both monoclonal and polyclonal gammopathies [15]. The removal of FLC reported in other recent studies has been around 84% for kappa [22] and 32.2–49.5% [23] or 69.3% [22] for lambda chains. Although other groups have shown a reduction of serum FLC with hemodiafiltration with ultrafiltrate regeneration [22–24], our study is the first that analyzes the concentration of FLC and albumin in both, blood and ultrafiltrate. The FLC reduction ratio obtained in the ultrafiltrate was greater than in blood. It was demonstrated that albumin was not adsorbed by the resin cartridge and was reinfused before the second chamber. Thus, the Super High Flux with polyphenylene membrane allowed the passage of FLC (especially kappa), without loss of albumin. According to the cut off of this first filter, which is the limiting factor for the FLC’s blood extraction and on the basis of the dimeric composition of lambda chains, it can be assumed that in hemodiafiltration with ultrafiltrate regeneration the kappa chains extraction was twice than lambda. The membrane permeability for a solute mainly depends on the molecular weight and it is modified by the shape of the solute, ionic charge and hydrodynamic radius. The molecular weight cut-off is defined as the molecular weight where the sieving coefficient is 0.1 [25, 26]. Based on this definition and using the information previously reported it is assumed that the cut-off of Supra HFR is close to albumin molecular weight (60 kDa) since the sieving coefficient is 0.11. In fact, in our study a small amount of albumin was detected on the UF that was reinfused into the extracorporeal circuit, since it was nor adsorbed by the resin. This is the reason why the loss of albumin remained very low, only 0.015% at the end of the procedure. Therefore if the sieving coefficient of Supra HFR for albumin is 0.11, the permeability for lambda free light chain (molecular weight 45 KDa) should be high enough to produce an appreciable reduction in its serum concentration. Other authors have observed that the use of filters with limited cut-off is capable of reducing circulating levels of FLC. It has been reported that the HFR17 a filter with a cut-off of 35 KDa was able to reduce serum levels of lambda light chains by 30.3 ± 2.9% [21].

The polymethylmethacrylate (PMMA) membranes have obtained satisfactory results in terms of light chain removal [27], but the adsorption process is limited by the saturation of the membrane. Standard PMMA hemodialysis (PMMA membrane BK 2.1 m^2^ Toray®) was compared with an enhanced adsorption dialysis, which involves PMMA dialyzer replacement after 2 h. The reduction of FLC was greater in enhanced adsorption than standard PMMA dialysis: 31% for kappa and 53% for lambda vs. 22% for kappa and 21% for lambda. Thus, to maintain FLC reduction and avoid membrane saturation, it is required to replace the dialyzer during the session [28].

The HCO membranes have a large pore (cut-off of 45–60 kD), allowing the filtration of both FLC [5, 29, 30]. The removal of FLC with HCO membrane is 66–69% of kappa and 71–90% of lambda [8, 31]. The procedure is more effective if applied promptly after an early diagnosis and treatment of the MM [5, 32]. Hutchison et al. showed that the factors which predicted non need of further dialysis were the degree of FLC reduction at days 12 and 21 as well as the time to initiating HCO-HD [9]. Moreover, Sens et al. reported a significant recovery of renal function if the FLC reduction rate was > 75% after 12 days of the initiation of chemotherapy and PMMA [33]. After 12 and 21 days from the beginning of chemotherapy and extracorporeal removal of FLC, the RR of FLC was 44.2 to 45.6% for kappa and 40.7 to 41.5% for lambda; with this reduction in FLC 33.3% recovered renal function and 44.4% required maintenance dialysis at the end of follow-up. Furthermore, in a recent randomized clinical trial involving 98 patients with myeloma cast nephropathy on chemotherapy, the use of HCO hemodialysis compared with conventional hemodialysis did not result in a significant benefit with respect to hemodialysis dependence at 3 months [12]. Later, Hutchison et al. shown that HCO hemodialysis was not associated with an improvement in renal recovery as compared with to High Flux- hemodialysis in patients with a new diagnosis of MM and myeloma cast nephropathy who received modified-dose bortezomib-containing chemotherapy although in both groups there was a sustained early reduction in serum FLC levels [13].

Other advantages of the hemodiafiltration with ultrafiltrate regeneration are: First, there was no decrease of albumin in blood and there was no adsorption of albumin to the resin; thus, albumin replacement is not required. That is a relevant advantage of this technique because combine hemodialysis with apheresis in a unique session of reasonable length, without the need for albumin replacement and the potential loss of other proteins of the immune system with a danger of infectious complications in subjects at a high risk due to the background haematologic disease. Second, in addition to avoiding further deposition of FLC to reduce the renal toxicity burden, it allows a rapid clearance of FLC that can be harmful elsewhere outside of the kidney, particularly amyloidogenic lambda chains for the heart. Third, there were no complications associated with the technique. It should be noticed that only 22.2% of the patients died during the first year and the cause of death was septic shock secondary to comorbidities associated to MM itself or its treatment (infections, immunosuppression, autotransplantation of hematopoietic cells and others); another two patients also died during the follow-up, so total mortality during the entire follow-up was 33.3% (patients 1, 4 and 5). Others have reported an increased mortality during the first year in MM patients undergoing hemodialysis treatment [34]. Yadav et al. shown that the median time to death in these patients was 5.1 months [35]. It is important to highlight that an early initiation of hemodialysis and prompt hemodialysis may improve the outcome [34].

In our study, renal recovery was achieved in two patients with kappa MM and in one patient with lambda MM. In a single-center study, renal recovery was associated with effective treatment of MM and a sustained reduction in the concentration of the involved FLC clone. The majority of patients who recover renal function have no further need for dialysis [35]. It has been described that the median overall survival in people who recovered renal function was 62.4 [36] – 64.1 [35] months. Joseph et al. recently published that renal recovery is inversely associated with more aggressive malignancies, with a worse proteinuria and with a history of high-dose therapy combined with autologous stem cell transplantation [3]. The published data on renal recovery using PMMA is not uniform. Sens et al. showed that an intensive hemodialysis with PMMA (6 h session requiring two PMMA filters (BK-F 2.1 m^2^ dialyzers) per session) resulted in a high rate of renal recovery (71%) and survival (62% alive at 24 months) [33]. However, Hudier et al. after intensive hemodialysis with PMMA, but using only one filter per session, did not observed a clear benefit on renal function as compared with standard PMMA hemodialysis; renal recovery rate was 38% after intensive hemodialysis as compared with 35% in patients receiving standard hemodialysis [37]. Therefore, hemodiafiltration with ultrafiltrate regeneration achieves a greater and maintained reduction of FLC and has promising results on renal recovery as compared to other adsorptive techniques (PMMA).

## Conclusions

In conclusion, hemodiafiltration with ultrafiltrate regeneration is a safe technique and provides a significant and sustained reduction of FLC in patients with AKI secondary to MM. The adsorptive capacity is maintained throughout the session and there is no loss of albumin. If hemodiafiltration with ultrafiltrate regeneration is used early in combination with effective chemotherapy, renal recovery is significant, regardless of the predominant type of light chains.

## Data Availability

The datasets used and/or analyzed during the current study are available from the corresponding author on reasonable request.

## Abbreviations

AKI: Acute Kidney Injury
FLC: Free light chains
HCO: High-Cut-Off
Supra HFR: Hemodiafiltration with ultrafiltrate regeneration
MM: Multiple myeloma
PMMA: Polymethylmethacrylate
RRs: Reduction ratio per session
UF: Ultrafiltrate
